# MoS_2_/S@g-CN Composite Electrode for L-Tryptophan Sensing

**DOI:** 10.3390/bios13110967

**Published:** 2023-11-02

**Authors:** Theophile Niyitanga, Aarti Pathak, Archana Chaudhary, Rais Ahmad Khan, Haekyoung Kim

**Affiliations:** 1 School of Materials Science and Engineering, Yeungnam University, Gyeongsan 38541, Republic of Korea; 2 Department of Chemistry, Medi-Caps University, Indore 453331, Madhya Pradesh, Indiaarchana@iiti.ac.in (A.C.); 3 Research and Development Cell, Indian Institute of Technology, Indore 453552, Madhya Pradesh, India; 4 Department of Chemistry, College of Science, King Saud University, Riyadh 11451, Saudi Arabia

**Keywords:** MoS_2_/S@g-C_3_N_4_, L-tryptophan, sensors, cyclic voltammetry, differential pulse voltammetry (DPV)

## Abstract

L-tryptophan (L-TRP) is an essential amino acid responsible for the establishment and maintenance of a positive nitrogen equilibrium in the nutrition of human beings. Therefore, it is vital to quantify the amount of L-tryptophan in our body. Herein, we report the MoS_2_/S@g-CN-modified glassy carbon electrode for the electrochemical detection of L-tryptophan (L-TRP). The MoS_2_/S@g-CN composite was successfully synthesized using an efficient and cost-effective hydrothermal method. The physical and chemical properties of the synthesized composite were analyzed using powder X-ray diffraction (PXRD), scanning electron microscopy (SEM), X-ray photoelectron spectroscopy (XPS), and energy-dispersive X-ray analysis (EDX). The crystallite size of the composite was calculated as 39.4 nm, with porous balls of MoS_2_ decorated over the S@g-CN surface. The XPS spectrum confirmed the presence of Mo, S, O, C, and N elements in the sample. The synthesized nanocomposite was further used to modify the glassy carbon (GC) electrode (MoS_2_/S@g-CN/GC). This MoS_2_/S@g-CN/GC was used for the electrochemical detection of L-TRP using cyclic voltammetry (CV) and differential pulse voltammetry (DPV) techniques. For the purpose of comparison, the effects of the scanning rate and the concentration of L-TRP on the current response for the bare GC, S@g-CN/GC, MoS_2_/GC, and MoS_2_/S@g-CN/GC were studied in detail. The MoS_2_/S@g-CN-modified GC electrode exhibited a rational limit of detection (LoD) of 0.03 µM and a sensitivity of 1.74 µA/ µMcm^2^, with excellent stability, efficient repeatability, and high selectivity for L-TRP detection.

## 1. Introduction

L-tryptophan is an important amino acid found in the human body that was discovered in the early 1900s from casein, which is an essential protein found in milk. L-tryptophan cannot be synthesized in the human body and is found in an inadequate amount in vegetables and fruits, but has considerable significance for human beings [1]. It is an important element of our diet, and is mainly found in protein-rich foods, viz., milk, cheese, eggs, meat, chicken, fish, nuts, and soy [2]. L-tryptophan is also essential to establish and maintain a positive nitrogen equilibrium in the nutrition of human beings [3,4]. Besides being a crucial element of protein, it also takes part in the synthesis of niacin. Niacin is a precursor for two of the most essential hormones (i.e., melatonin and serotonin) in our body [5]. Melatonin and Serotonin hormones function for the proper sleep & physiology, help in regulating body temperature & blood pressure, effects immune system, circadian rhythm, sexuality, and nutrition [6,7,8,9]. According to the World Health Organization (WHO), a human should consume 4 mg/kg per day of L-tryptophan. However, due to the malfunctioning of the metabolism of L-tryptophan, the generation of toxic waste occurs in the brain, leading to illusions and hallucinations. As a result of this, neurological dysfunction occurs [4,10,11]. So, it becomes essential to determine the levels of L-tryptophan in biological samples, food, and pharmaceutical samples [12,13].

Over the previous decade, scientists have focused on the design and development of analytical methods dedicated to the detection of biomolecules (L-tryptophan, ascorbic acid, dopamine, uric acid, etc.) present in living species. It is easy to detect them separately, but in the human body, different biomolecules coexist. Hence, it is a daunting task to detect them in a single run, as the physical and chemical properties of these biomolecules are different. Based on the literature survey, it can be noted that there are several methods for the analysis of L-tryptophan that use chromatography [14,15,16,17] and spectroscopy [18,19,20] techniques. However, there are several problems associated with these methods, viz., being difficult to handle, high costs, and the need for qualified personnel [9]. In contrast to these analytical techniques, nonenzymatic electrochemical detection techniques may be advantageous for the detection of L-tryptophan, as, in these techniques, the oxidation potential of the species is recorded and analyzed. These electrochemical detection techniques (cyclic voltammetry, differential pulse voltammetry, and linear sweep voltammetry) are highly sensitive and selective, provide a rapid response, are cost-effective, and are less time-consuming [9,21]. 

In the electrochemical detection method, the working electrode, glassy carbon/screen-printed electrodes, etc., can be chemically modified using various materials. In these chemically modified electrodes (CMEs), electrons are transported between a solution of the analyte and the substrate electrode by redox-active sites, generally with a significant decline in the activation overpotential. The materials, which are used for the modification of the electrode, possess some unique properties that provide enhanced electrocatalytic activity for the detection of L-tryptophan. These modified electrodes are less prone to surface fouling and oxide formation compared to the bare electrodes. Nanomaterials, metal complexes, conducting polymers (CPs), ionic liquids (ILs), and composite materials are used for the modification of these electrodes. Other trends, like combining different modified materials, are also being followed extensively. These modified materials are a combination of a few well-known materials, such as carbon, metal, or metal-oxide nanoparticles [22].

Molybdenum disulfide (MoS_2_), which is one of the very important members of the transition-metal dichalcogenides (TMDs) family, has a graphene-like 2D structure and can be used for a wide range of applications, viz., photocatalysts, biosensors, sensors, transistors, and supercapacitors [15,17]. In the crystal of MoS_2_, the Mo atom is attached to two S atoms (S-Mo-S) by a covalent bond, forming a layer. These layers in the 2D structure are stacked together via weak van der Waals interactions, forming several layers of MoS_2_ [15,17,18,19]. The 2D MoS_2_ is also used as a transducer modifier material in many sensors and biosensors [9,20,23,24,25].

Graphitic carbon nitride (g-C_3_N_4_), a carbon-based material, finds a wide number of applications in photocatalysis, catalysis, sensing, etc., as it is cost-effective to fabricate and shows good adaptable behavior [26,27,28]. g-C_3_N_4_ is a graphene-like functional material that shows unique features, viz., an interesting electronic band structure, a low fabrication cost, and high stability [28]. However, the practical applications of g-C_3_N_4_ are limited due to its low conductivity, weak π−π-conjugated stacked structure, and low surface area [26,27]. In order to enhance the performance of g-C_3_N_4_, it is modified with metal/metal oxides, nonmetals, graphitic carbon nitride/nanotubes and their hybrids, and so on [26,27,28,29,30,31,32,33,34,35,36,37,38,39,40,41,42,43,44,45,46,47,48,49]. 

In previous years, it was reported that combining the two materials may boost the electrocatalytic properties of the hybrid composite materials. In this connection, Wang et al. [28] reported the fabrication of a nitrite sensor using a flower-like MoS_2_-decorated g-C_3_N_4_ composite. In another report, Nehru et al. [29] also synthesized a MoS_2_/g-C_3_N_4_ composite and fabricated a sensor for the detection of vanillin using voltametric methods. This showed that the preparation of MoS_2_ with g-C_3_N_4_ may boost their electrocatalytic properties, and that the presence of synergism between MoS_2_ and gCN is responsible for the enhanced electrochemical sensing performance of the developed sensors. Thus, it will be of great significance to further develop the MoS_2_- and g-C_3_N_4_-based electrochemical sensors for the determination of L-TRP. 

Herein, we report the “MoS2/S@g-CN Composite Electrode for L-Tryptophan Sensing”.

## 2. Materials and Methods

### 2.1. Chemicals

Sodium molybdate dehydrate (≥98%) and thiourea (ACS reagent, ≥99.0%) were purchased from SigmaGlycine (≥99%), l-cysteine (97%), l-methionine (reagent grade, ≥98%), tyrosine (reagent grade, ≥98%), and l-proline (ReagentPlus, ≥99%), leucine, L-tryptophan (reagent grade, ≥98%), and phenylalanine (reagent grade, ≥98%) were bought from Merck. Hydrazine hydrate (64–65%, reagent grade, 98%), catechol (≥99%), urea (ACS reagent, 99%), ascorbic acid, glucose, and hydrogen peroxide (34.5–36.5%) were purchased from Sigma (Chennai, India) Dopamine (98%) and para-nitrophenol (98%) were purchased from SRL. All of the chemicals and reagents were used as received.

### 2.2. Synthesis of MoS_2_/S@g-C_3_N_4_

Initially, we synthesized MoS_2_ using the hydrothermal method. Typically, 430 mg of sodium molybdate was dissolved in 35 mL of DI water. In another beaker, 600 mg thiourea was dissolved in 10 mL of DI water. The prepared aqueous solution of thiourea was slowly added to the aqueous sodium molybdate solution and stirred for 30 min at 500 rpm. This reaction solution was poured in a Teflon reactor (100 mL capacity), which was covered with a stainless steel autoclave. The autoclave was kept at 200 °C for 24 h in a muffle furnace. The black solid precipitate was collected and washed with DI water and ethanol three times to remove the unreacted or residual particles. The washed product was dried at 60 °C for 12 h and characterized as MoS_2_. Sulfur-doped graphitic carbon nitride (S@g-CN) was obtained simply through the calcination of thiourea. In brief, thiourea was kept in a heat-resistant crucible, which was heated at 550 °C resulting in S@g-CN. For synthesizing the MoS_2_/S@g-CN composite, 50 mg of the above-prepared S@g-CN was dispersed in 10 mL of DI water and sonicated for 20 min. On the other hand, an aqueous solution of thiourea (600 mg/10 mL) was slowly added to the aqueous solution of sodium molybdate (430 mg/35 mL), and, finally, S@-g-CN dispersion was added to this reaction mixture. The prepared reaction solution was poured in the Teflon reactor (100 mL capacity) and this reactor was covered with a stainless steel autoclave. The autoclave was kept at 200 °C for 24 h in a muffle furnace. The black solid precipitate was collected and washed with DI water and ethanol three times to remove the unreacted or residual particles. The washed product was dried at 60 °C for 12 h. The obtained product was designated as MoS_2_/S@g-CN composite.

### 2.3. Instrumental Characterization

The hydrothermally synthesized MoS_2_ and MoS_2_/S@g-CN and S@g-CN samples were characterized by employing a RINT 2500 V powder X-ray diffractometer (Cu-Ka irradiation; λ = 1.5406 Å), Rigaku, Japan. The microstructures of the synthesized samples were evaluated on a Supra 55 Zeiss Oxford scanning electron microscope (SEM). The elemental composition of the synthesized samples were checked using energy-dispersive X-ray spectroscopy (EDX) on an Oxford X-max Aztec spectroscope. The X-ray photoelectron spectroscopic (XPS) spectrum of the composite was collected on a Fisher Scientific spectroscope (Thermo Scientific™ ESCALAB Xi+™ XPS Microprobe, Waltham, MA, USA). The application part (electrochemical sensing curves) was carried out on a CH instrument comprising a three-electrode system. The GC, Ag/AgCl, and Pt were used as working, reference, and counter electrodes, respectively, for the measurements of the electrochemical results.

### 2.4. Fabrication of L-Tryptophan Sensor

The GC electrode was modified with the above-prepared MoS_2_/S@g-CN composite and designated as a MoS_2_/S@g-CN/GC electrode. In detail, MoS_2_/S@g-CN was dispersed in DI water (4 mg electro-catalyst in 3 mL DI water + 0.1% nafion) via ultrasonication for 15 min. Furthermore, an appropriate amount of the above electro-catalyst was drop-wise deposited on a bare glassy carbon (GC) electrode surface. This modified GC electrode was further kept in the air for several hours to allow it to dry completely. Control experiments were also performed, and for that purpose, the same amount of MoS_2_ or S@g-CN was deposited on a bare GC electrode surface. The modified electrodes are designated as MoS_2_/GC and S@g-CN/GC electrodes. For electrochemical sensing experiments, three electrode systems were used. Ag/AgCl was adopted as the reference electrode and a platinum electrode was used as the counter electrode. The GC and modified GC electrodes were used as working electrodes.

## 3. Results and Discussion

### 3.1. Characterization

**Figure 1:**
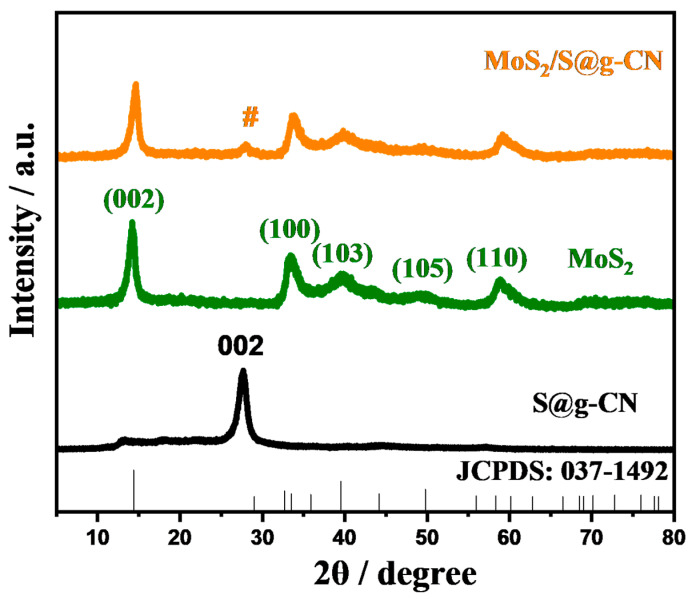
XRD patterns of the synthesized samples (S@g-CN, MoS_2_, and MoS_2_/S@g-CN) (Color Code: Black= S@g-CN; Green= MoS_2_; Orange = MoS_2_/S@g-CN; # = suggests the generation of Mos_2_/S@g-CN composite).

In order to characterize the generated phase in the samples of S@g-CN_,_ MoS_2_, and composite of MoS_2_/S@g-CN, PXRD patterns were recorded in the 2θ range of 5–80° and are shown in Figure 1. In the XRD pattern of S@g-CN, the characteristic diffraction plane is observed at 27.64°, denoting the (002) plane of S@g-CN. This (002) plane is attributed to the interlayer stacking reflection, which occurs because of the conjugated triazine aromatic layers [40]. A slight shift in the peak position (JCPDS card no. 87–1526) may be due to the sulfur-doping in g-CN. In the PXRD of MoS_2_, five diffraction peaks are observed at 2θ of 14.35°, 35.12°, 39.78°, 49.73°, and 58.65°, which correspond to the (002), (100), (103), (105), and (110) diffraction planes, respectively, of MoS_2_. Here, MoS_2_ crystallizes in a hexagonal crystal system in the P63/mmc space group. The PXRD patterns of MoS_2_ resemble the JCDPS card No. 037-1492. The PXRD of the MoS_2_/S@g-CN composite demonstrated similar diffraction planes to the (002), (100), (103), (105), and (110) diffraction planes of MoS_2_. However, a diffraction peak in S@g-CN also appeared, which suggests the formation of MoS_2_/S@g-CN composite. No additional peaks were observed in the P-XRD patterns of S@g-CN_,_ MoS_2_, or MoS_2_/S@g-CN, suggesting that the fabricated samples are phase-pure.

For calculating the crystallite size of the MoS_2_/S@g-CN composite, the Debye Scherrer equation (Equation (1)) was used.

D = K·λ/βCosθ(1)

where D = crystalline size;

K = Scherrer constant (0.98);
λ = wavelength (1.54 Å);
β = full width at half maximum (FWHM).

The crystallite size of the MoS_2_/S@g-CN composite was calculated to be 39.4 nm.

**Figure 2:**
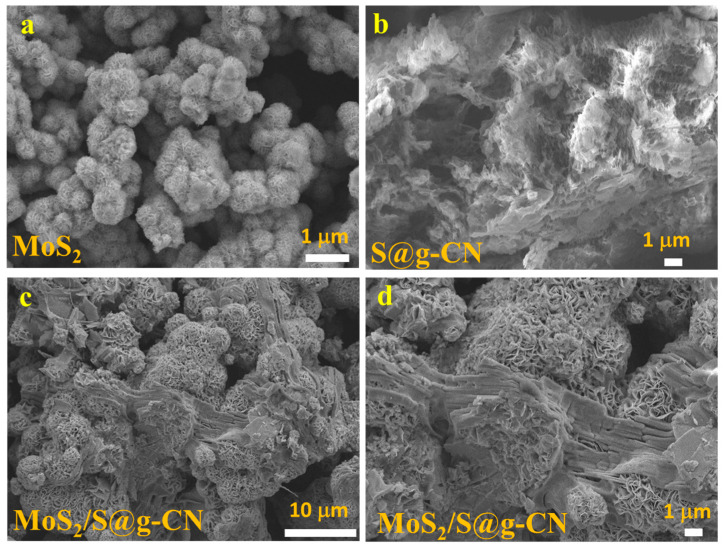
SEM image of the synthesized MoS_2_ (**a**), S@g-CN (**b**), and MoS_2_/S@g-CN (**c**,**d**).

In electrochemical and optoelectronic applications, the surface morphology and structural properties of the material to be used play significant roles. Therefore, for evaluating the surface morphologies of S@g-CN, MoS_2_, and MoS_2_/S@g-CN composite, SEM images were recorded. The SEM images of all of the samples are shown in Figure 2. Figure 2a demonstrates the surface morphology of MoS_2_, exhibiting porous ball-like structures at the scale of 1 µm. Stable, porous-material-modified electrodes are advantageous as there is a higher level of interaction between electrolytes and the active surface of the material. This results in improved electrochemical reactions, shorter diffusion lengths, and reduced Ohmic, polarization, and concentration resistances. In Figure 2b, the SEM image of S@g-CN is shown, confirming the sheet-like structure of S@g-C_3_N_4._ Figure 2c,d exhibit SEM images of MoS_2_/S@g-CN at two different scales, 10 µm (Figure 2c) and 1 µm (Figure 2d). Both images show the decoration of MoS_2_ balls over the S@g-CN sheet. The SEM images indicate that MoS_2_ and S@g-CN are in close proximity to each other, confirming the formation of the MoS_2_/S@g-CN composite.

**Figure 3:**
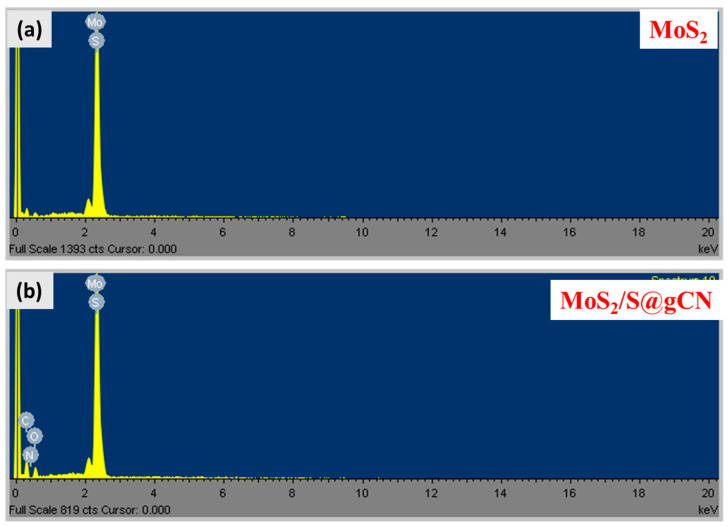
EDX spectra of the MoS_2_ (**a**) and MoS_2_/S@g-CN (**b**).

Further, to determine the phase purity of the samples, it is essential to identify the elements present in it. Hence, the elemental composition of MoS_2_ and MoS_2_/S@g-CN composite were determined using energy-dispersive X-ray analysis (EDX). The EDX of both samples are presented in Figure 3a,b. In the EDX of MoS_2_, peaks for Mo and S elements are observed, whereas the EDX of the MoS_2_/S@g-CN composite exhibits peaks for Mo, S, C, O, and N. The absence of any additional peak in the EDX of both samples further indicates that the fabricated MoS_2_/S@g-CN composite and MoS_2_ are highly pure in nature.

**Figure 4:**
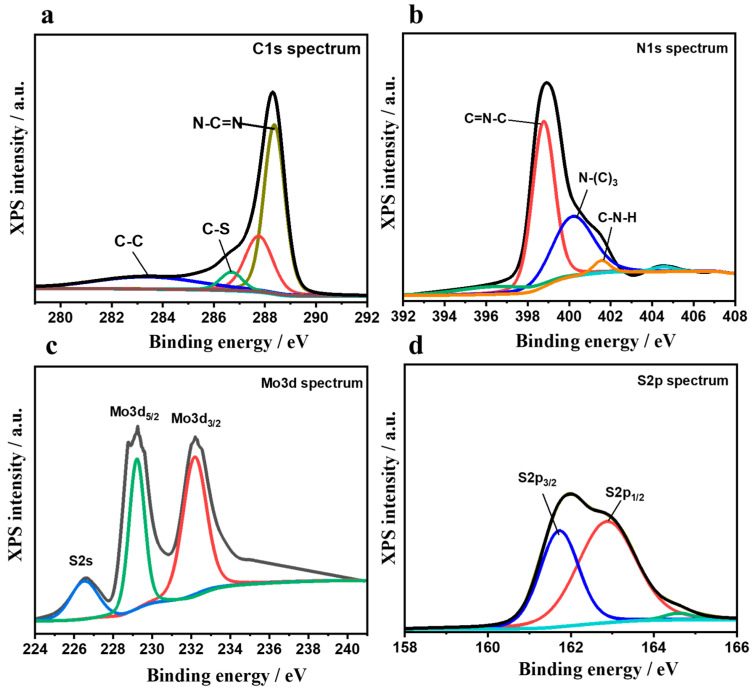
XPS study: high resolution XPS spectra of C1s (**a**), N1s (**b**), Mo3d (**c**), and S2p (**d**) of the synthesized MoS_2_/S@g-CN composite.

Besides EDX, we also performed X-ray photoelectron spectroscopy (XPS) for detecting the elements present in MoS_2_/S@g-CN and their respective oxidation states. The spectra of the XPS study are shown in Figure 4a–d. In the high-resolution C1 spectrum (Figure 4a), peaks at 283.6 eV, 286.6 eV, and 288.3 eV may refer to sp^3^-bonded carbon in the C-C bond, C-S bond, C-O, and tertiary carbon N-C=N, respectively. In the N1 spectrum, peaks at 398.7 eV, 400.2 eV, and 401.5 eV are attributed to C=N-C, tertiary nitrogen atom (N-C_3_), and C-N-H, respectively (Figure 4b). In the high-resolution spectrum of Mo3d, two deconvoluted peaks centered at 229.1 and 232.1 eV correspond to the Mo^4+^ 3d_5/2_ and Mo^4+^ 3d_3/2_ oxidation states, respectively. These deconvoluted peaks are in accordance with the expected oxidation state of MoS_2_, as shown in Figure 4c [42]. Along with this, a small peak was observed at 226.5 eV, which may be ascribed to the S2s spectrum in MoS_2_. In the high-resolution S2p spectrum, two peaks at 161.7 and 162.9 eV were observed, which are ascribed to S2p_3/2_ and S2p_1/2_, respectively. The peaks of S2p_3/2_ and S2p_1/2_ confirm the presence of sulfide (S^2−^) in the composite. The obtained XPS results are in accordance with the results of XRD, SEM, and EDX, strongly suggesting that the MoS_2_/S@g-CN composite could be successfully fabricated using the hydrothermal method [50].

### 3.2. Sensing Behavior of MoS_2_/S@g-C_3_N_4_-Modified Electrode

**Figure 5:**
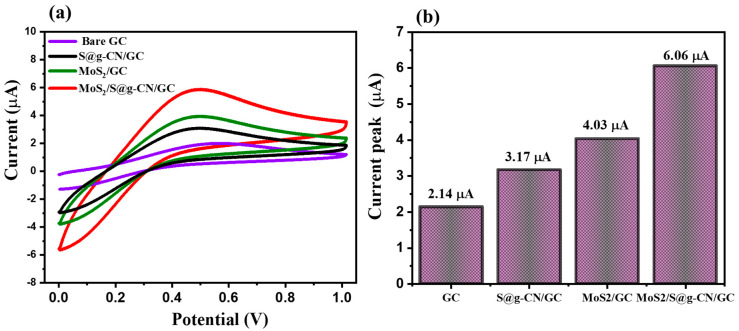
CV patterns (**a**) and peak current values (**b**) of the bare GC, S@g-CN, MoS_2_/GC, and MoS_2_/S@g-CN/GC in the presence of 25 µM L-TRP at 0.05 V/s in 0.1 M PBS of pH 3.

For evaluating the electrochemical sensing performance of S@g-CN, MoS_2_, and MoS_2_/S@g-CN composite, all three fabricated samples were deposited onto glassy carbon electrodes. For comparative study, these modified electrodes were used for the electrochemical detection of L-TRP along with the bare glassy carbon (GC) electrode. All of the CV graphs were recorded in the presence of 25 µM of L-TRP in 0.1 M PBS buffer (the pH of the PBS buffer was 3.0) along with the applied scan rate of 0.05 V/s, in the potential range of −0.1 to 0.1 V (Figure 5a). The peak for the oxidation potential of L-TRP could be observed at 0.42 V with a low electrocatalytic current response of 2.14 µA for the GC electrode [51]. For the S@g-CN-modified GC electrode, the electrocatalytic current response increases slightly, i.e., 3.17 µA, which is 4.03 µA for the MoS_2_-modified GC electrode. When this GC electrode was modified with MoS_2_/S@g-CN_,_ the value of the current response increased drastically (approximately threefold) compared to the bare GC electrode, i.e., 6.06 µA (Figure 5a,b).

**Figure 6:**
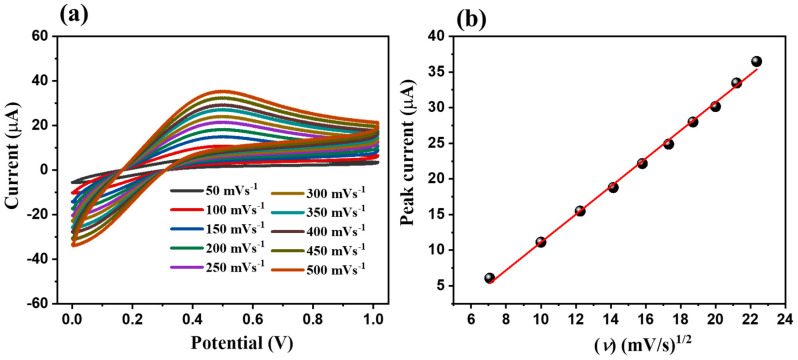
CV patterns of the MoS_2_/S@g-CN/GC in the presence of 25 µM L-TRP at different scan rates (0.05–0.5 V/s) in 0.1 M PBS of pH 3 (**a**) and corresponding calibration curve between peak current and square root of scan rate (**b**).

Various scan rates may affect the results of electrochemical sensing differently; thus, the impact of increased applied scan rates to evaluate the electrochemical ability of MoS_2_/S@g-CN/GC has also been studied. For executing these experiments, the concentration of L-TRP was fixed at 25 µM, in 0.1 M PBS at the acidic pH of 3.0. The applied scan rates were increased from 0.05 V/s to 0.5 V/s. In Figure 6a, the CV graphs for these experiments are shown. The results of these experiments show that on increasing the scan rate from 0.05 V/s to 0.5 V/s, the current response improves. The calibration curve of the current responses vs. the square root of the applied scan rates is shown in Figure 6b. The shown calibration plot suggests that there is a linear increase in the current response with the increasing applied scan rate (R^2^ = 0.99).

**Figure 7:**
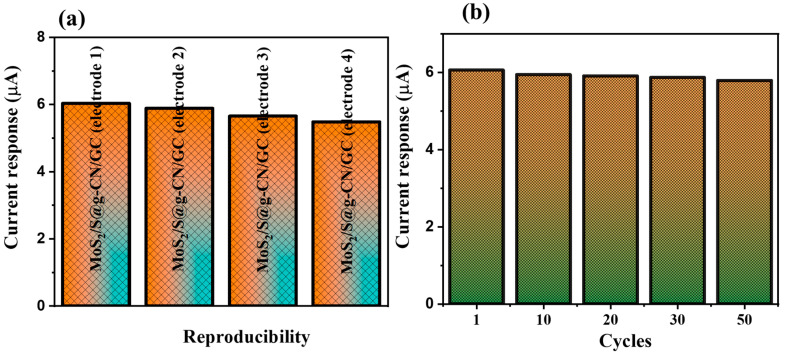
Reproducibility (**a**) and repeatability (**b**) study for MoS_2_/S@g-CN/GC for 25 µM L-TRP at 0.05 V/s in 0.1 M PBS at pH 3.

Reproducibility and repeatability are two very significant parameters when considering using an electrochemical sensor in real life. We recorded the repeatability tests of the designed MoS_2_/S@g-CN/GC electrode for the detection of L-TRP by recording fifty consecutive CV cycles. For this experiment, the concentration of L-TRP was fixed at 25 µM and the scan rate was taken as 0.05 V/s. In the reproducibility study of L-TRP, the concentration was fixed at 25 µM in 0.1 M PBS at an applied scan rate of 0.05 V/s. The obtained results showed good reproducibility. The reproducibility results are summarized in Figure 7a and Figure S2. The consecutive CV graphs of the MoS_2_/S@g-CN/GC electrode for L-TRP detection were recorded and are displayed in Figure S3. The obtained results exhibited the presence of good stability and repeatability up to 50 cycles (Figure 7b and Figure S3).

**Figure 8:**
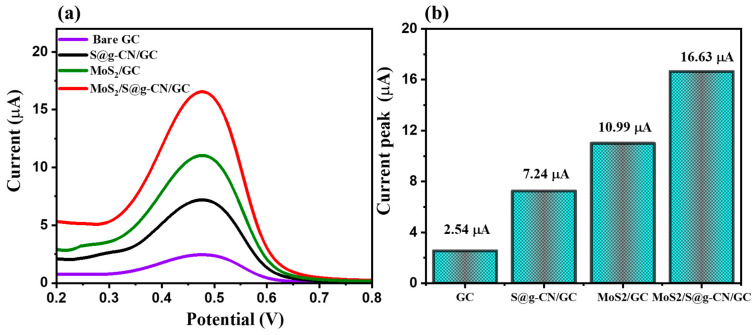
DPV patterns (**a**) and peak current values (**b**) of bare GC, S@g-CN, MoS_2_/GC, and MoS_2_/S@g-CN/GC in the presence of 25 µM L-TRP in 0.1 M PBS of pH 3 at 0.05 V/s.

According to recent literature reports, differential pulse voltammetry (DPV) is considered a more efficient and sensitive electrochemical detection technique than cyclic voltammetry (CV) or linear sweep voltammetry (LSV). Therefore, DPV scans were applied for the electrochemical detection of L-TRP. Here, L-TRP was detected on the bare GC, S@g-CN/GC, MoS_2_/GC, and MoS_2_/S@g-CN/GC electrodes (Figure 8). The DPV experiments were performed in the presence of 25 µM L-TRP using 0.1 M PBS buffer at pH = 3.0 with the applied scan rate of 0.05 V/s and in the potential range of 0.2 to 0.8 V (Figure 8a). The current response, as observed via the DPV graph, for bare GC is 2.54 µA, for S@g-CN/GC is 7.24 µA, for MoS_2_/GC is 10.99 µA, and for MoS_2_/S@g-CN/GC is 16.63 µA. It was noticed that the current response for the MoS_2_/S@g-CN/GC increased almost sixfold compared to that of the bare GC. The efficient results of the current response of differential pulse voltammetry (DPV) suggest that the DPV technique is comparatively better to use for further electrochemical sensing applications.

**Figure 9:**
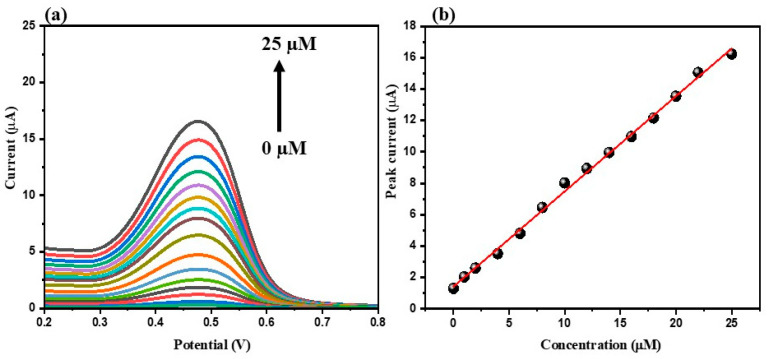
DPVs (**a**) of MoS_2_/S@g-CN/GC for various concentrations (0, 0.03, 1, 2, 4, 6, 8, 10, 12, 14, 16, 18, 20, 22, and 25 µM) of L-TRP at 0.05 V/s in 0.1 M PBS of pH 3. Corresponding calibration curve (**b**) between current responses versus concentration of L-TRP.

We also studied the variation in the current response by varying the concentration of L-tryptophan (0, 0.03, 1, 2, 4, 6, 8, 10, 12, 14, 16, 18, 20, 22, and 25 µM) to determine the electrochemical detection ability of the MoS_2_/S@g-CN/GC by using DPV graphs. The buffer solution used was 0.1 M PBS at the applied scan rate 0.05 V/s and pH at 3. The pH of the solution affects the performance of the fabricated electrodes. Thus, we also optimized the pH of the analyte solution. The DPVs of MoS_2_/S@g-CN/GC for 25 µM L-TRP at 0.05 V/s in 0.1 M PBS of different pH levels (1, 3, 5, 7, and 9) were recorded. The observations suggested the presence of a high current response at pH 3. The obtained results are summarized in Figure S1. The DPV graph depicts an increase in the current response with the increasing L-TRP concentration (Figure 9a). An increasing linear slope was observed with the increasing concentration for the calibration curve of the current response vs. concentration of L-TRP, as depicted in Figure 9b with R^2^ = 0.99.

**Figure 10:**
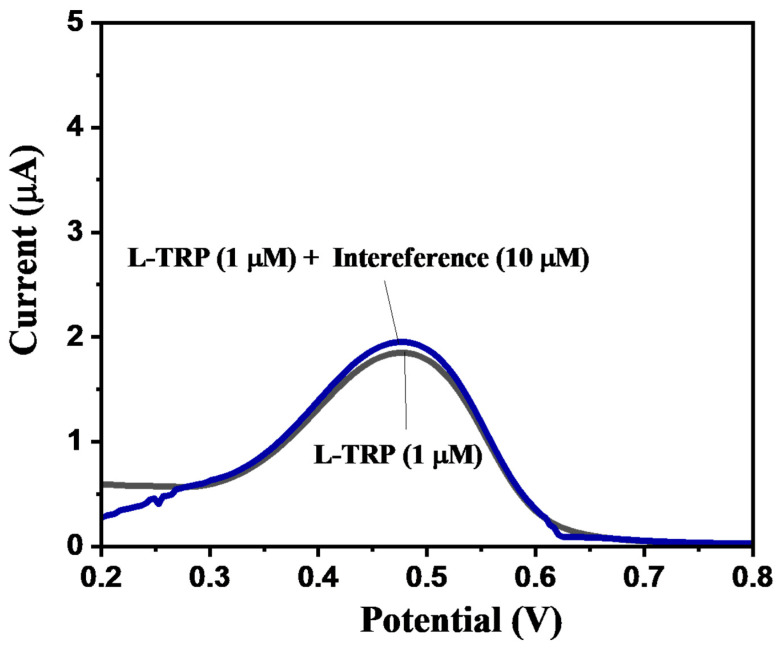
Selectivity study: DPV of MoS_2_/S@g-CN/GC for 1 µM L-TRP and 1 µM L-TRP + interferences (10 µM; glycine, l-cysteine, l-methionine, tyrosine, l-proline, leucine, and phenylalanine, at 0.05 V/s in 0.1 M PBS of pH 3).

For the practical and real-life application of an ideal and efficient electrochemical sensor, selectivity in the presence of other species remains one of the most required criteria. In the presence of foreign species, an interfering atmosphere may be created, which might lead to the improper and inaccurate analysis of the desired analyte during the electrochemical experiment. Hence, in order to determine the selectivity of MoS_2_/S@g-CN/GC for L-TRP, we selected the DPV scan. In our first experiment, we recorded the DPV curve for L-TRP on a MoS_2_/S@g-CN-modified GC electrode with a concentration of L-TRP 1 µM (Figure 10). The DPV curve was recorded for 1 µM of L-TRP in the presence of different interfering molecules (glycine, l-cysteine, l-methionine, tyrosine, l-proline, leucine, and phenylalanine) at a concentration of 10 µM for the MoS_2_/S@g-CN-modified GC electrode. In these set of experiments, the concentration of the interfering species was set ten times higher than L-TRP, with 0.1 M of PBS buffer at pH 3.0, and an applied scan rate of 0.05 V/s. The selectivity test was also carried out using hydrazine, catechol, urea, dopamine, 4-nitrophenol, ascorbic acid, glucose, and hydrogen peroxide as interfering species, as shown in Figure S4. No significant change was observed and suggested good selectivity. It is noteworthy to mention here that very insignificant variations in the current response were observed in the presence of interfering species (glycine, l-cysteine, l-methionine, tyrosine, l-proline, leucine, and phenylalanine), indicating the higher selectivity of MoS_2_/S@g-CN for L-TRP even if other interfering species are present in the system.

In the present work, we determined the electrochemical sensing parameters (limit of detection = LOD and sensitivity) of the MoS_2_/S@g-CN/GC for the detection of L-TRP using Equations (2) and (3), as given below.

LOD = 3.3 × standard error or deviation/slope(2)

Sensitivity = slope/Area of the GC − 2(3)

The calculated LOD and sensitivity of MoS_2_/S@g-CN/GC for the sensing of L-TRP are displayed in Table 1 and compared with previous literature [1,9,52,53,54,55,56].

Deng et al. [1] reported the fabrication of a graphene-modified acetylene black paste electrode (GR/ABPE) to detect L-tryptophan in the presence of tyrosine. The synthesized sensor was characterized using the cyclic voltammetry (CV) technique and the results demonstrated that the GR/ABPE depicts the limit of detection (LOD) value of 0.06 µM. Yıldız et al. [9] report the synthesis of a practical sensor based on a pencil graphite electrode (PGE) for the detection of L-TRP in human urine samples. The physical and chemical parameters of the synthesized samples were studied using appropriate characterization techniques, and the electrochemical sensing studies were performed using differential pulse adsorptive stripping voltammetry (DPAdSV). Under optimum conditions, viz., the accumulation potential of 0.3 V, accumulation time of 5.0 s, and a pH of 3.0, the DPAdSV peak revealed a LOD value of 0.046 µM. The current response also increased with the increasing L-TRP concentration. Tasic et al. [13] fabricated a cheap, non-toxic, and convenient electrochemical sensor for the detection of L-tryptophan in milk and apple juice samples. The authors reused the graphite rod from the zinc carbon batteries in order to reduce and eliminate electronic waste from the surroundings. The nano sensor was further utilized to determine the current response and limit of detection (LOD) value of the sensor via differential pulse voltammetry (DPV). The results demonstrated the LOD value to be 1.73 µM with efficiently enhanced sensitivity. Sun et al. [17] reported the fabrication of a carbon electrode through the processing of carbon layers over Ta wire using the chemical vapor deposition method. The designed electrode was further used as an electrochemical sensor for the determination of L-tryptophan and other analytes present in a serum sample. The results revealed that the current response increased enormously with the increasing concentration of L-TRP, and the limit of detection (LOD) value was calculated to be 0.24 µM. The sensitivity of the sample also increased efficiently.

## 4. Conclusions

Herein, the synthesis of a MoS_2_/S@g-CN-modified GC electrode developed using a cost effective and less time-consuming hydrothermal method was reported for the electrochemical sensing of L-tryptophan (L-TRP). The physical properties of MoS_2_/S@g-CN were studied using appropriate characterization techniques exhibiting the nano, porous balls of MoS_2_ decorated over the surface of S@g-CN with high phase purity. The sensing application of MoS_2_/S@g-CN was also evaluated for the electrochemical detection of L-TRP using cyclic voltammetry (CV) and differential pulse voltammetry (DPV) techniques. The results of the electrochemical detection experiments showed that MoS_2_/S@g-CN/GC is a stable, selective electrochemical sensor for L-TRP, and may be used for real-life applications as well. MoS_2_/S@g-CN/GC exhibited good repeatability and reproducibility using the CV method. MoS_2_/S@g-CN/GC also possesses excellent anti-interfering properties and can be used as a selective sensor for L-TRP. The reasonably good detection limit of 0.03 µM was achieved for the determination of L-TRP via the DPV method. This work proposes the synthesis of a MoS_2_/S@g-CN composite and the fabrication of a MoS_2_/S@g-CN-based L-TRP sensor.

## Figures and Tables

**Table 1:** Comparison of electrochemical detection parameters with previous reports.

Electrode	LOD (µM)	Sensitivity (µA/ µMcm^2^)	References
MoS_2_/S@g-CN/GC	0.03	1.74	Present work
GR/ABPE	0.06		[1]
PGE	0.04	-	[9]
Graphite rod	1.73	-	[13]
Carbon electrode	0.24		[17]
MIP/CS/MWCNTs/GCE	0.5	-	[52]
GE/MIP/Ppy	16.6	-	[53]
TiO_2_-MWNT/GCE	0.52	-	[54]
ZnO/CPE	0.57	-	[55]
FeZnSn-TDHae/GCE	1.22	-	[56]

## Data Availability

Not applicable.

## Supplementary Materials

The following supporting information can be downloaded at: https://www.mdpi.com/article/10.3390/bios13110967/s1.
