# Supplementary material for: MoS2/S@g-CN Composite Electrode for L-Tryptophan Sensing
Source: Biosensors (Basel). 2023 Nov 2;13(11):967. doi: 10.3390/bios13110967 (PMC10669109; doi:10.3390/bios13110967)
Supplement: Supplementary file 1 [file biosensors-13-00967-s001.zip › biosensors-2629472-supplementary.pdf]

Figure S1 depicts the variation in the current response with respect to the change in pH value of theL-tryptophan (L-TRP) in the range from pH 1.0-9. The buffer solution taken was 0.1 M PBS with the scan speed of 0.05 V/s. It has been observed been observed that the current increases slightly when the pH is increased from 1 to 3 but then it eventually decreases with the increasing pH value of the L-TRP. The DPV graph reveals that the optimum current response has been observed when the pH of the L-TRP is 3.0 whereas the minimum amount of current response calculated is at pH value 9.

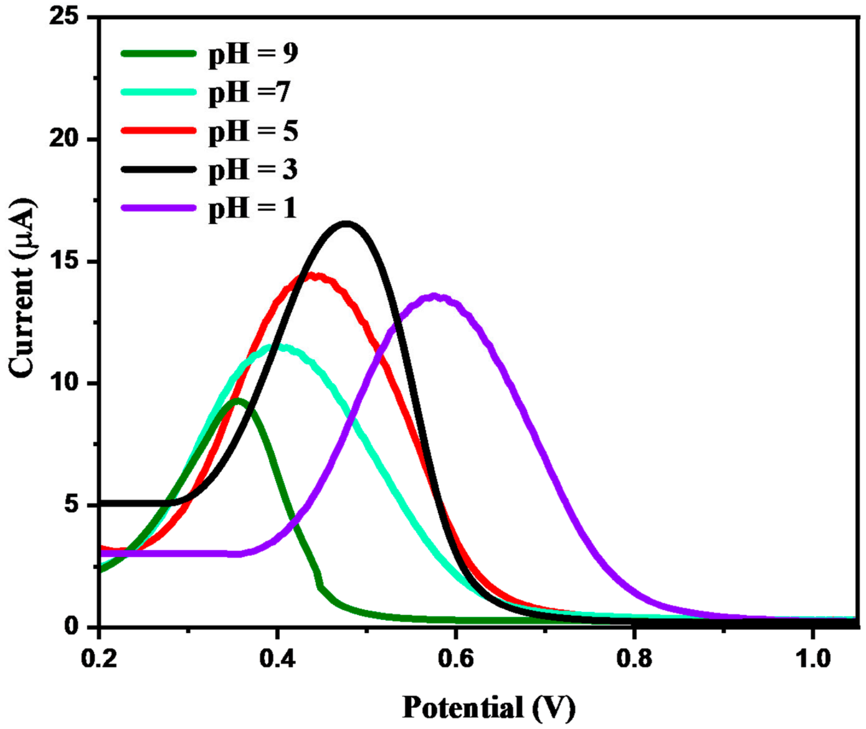

Figure S1. DPVs of MoS_2/S@g-CN/GC for 25 μM L-TRP at 0.05 V/s in 0.1 M PBS of different pH (1, 3, 5, 7, and 9).

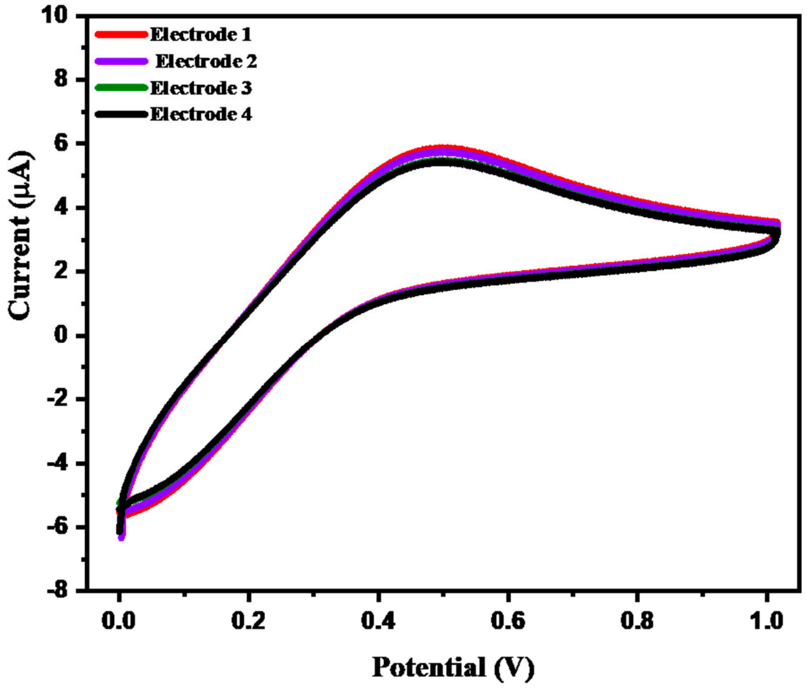

Figure S2. Reproducibility study (CV graphs) for MoS_2/S@g-CN/GC for 25 μM L-TRP at 0.05 V/s in 0.1 M PBS of pH 3.

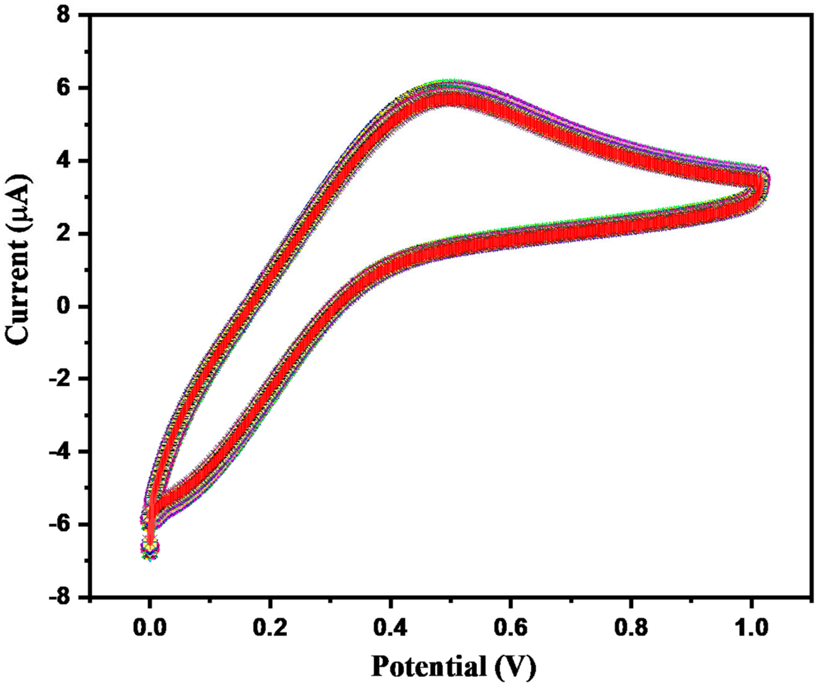

Figure S3. Repeatability study (50 consecutive CV graphs) for MoS_2/S@g-CN/GC for 25 μM L-TRP at 0.05 V/s in 0.1 M PBS of pH 3.

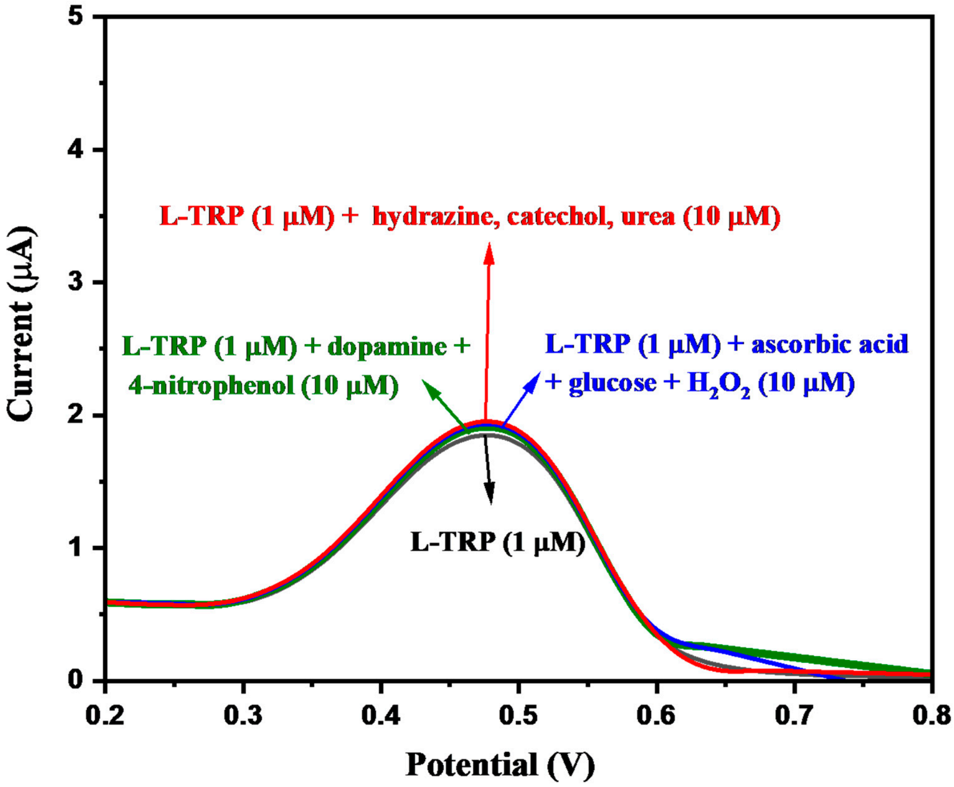

Figure S4. Selectivity study: DPV of MoS_2/S@g-CN/GC for 1 µM L-TRP and 1 µM L-TRP + interferences (10 µM; hydrazine, catechol, urea, dopamine, 4-nitrophenol, ascorbic acid, glucose and hydrogen peroxide) at 0.05 V/s in 0.1 M PBS of pH 3).
